# Physical activity levels, sleep quality and perceived stress in Chilean students: a cross-sectional study

**DOI:** 10.3389/fspor.2026.1789806

**Published:** 2026-04-28

**Authors:** Raúl Aguilera-Eguía, Héctor Fuentes-Barría, Yoselin Avilés-Santos, Felipe Aburto-Valenzuela, John Vergara-Erices, Vanessa Pizarro-Brante, Miguel Alarcón-Rivera, Exal Garcia-Carrillo

**Affiliations:** 1 Departamento de Salud Pública, Facultad de Medicina, Universidad Católica de la Santísima Concepción, Concepción, Chile; 2 Centro de Investigación en Medicina de Altura (CEIMA), Universidad Arturo Prat, Iquique, Chile; 3 Carrera de Kinesiología, Facultad de Medicina, Universidad Católica de la Santísima Concepción, Concepción, Chile; 4 Escuela de Ciencias del Deporte y Actividad Fisica, Facultad de Salud, Universidad Santo Tomas, Talca, Chile; 5 Department of Physical Activity Sciences, Faculty of Education Sciences, Universidad Católica del Maule, Talca, Chile; 6 Department of Physical Activity Sciences, Universidad de Los Lagos, Osorno, Chile

**Keywords:** perceived stress, physical activity, sex differences, sleep quality, University students

## Abstract

**Objective:**

To describe sleep quality, perceived stress, and physical activity levels, as well as to identify the associations between these variables in fourth-year Kinesiology students at the Catholic University of the Most Holy Conception**.**

**Methods:**

A descriptive cross-sectional study was conducted in 29 fourth-year Kinesiology students (16 women, 13 men; aged 21–27 years) from a Chilean university. Physical activity was assessed using IPAQ-SF, sleep quality with the PSQI, and perceived stress with the PSS-14. Group comparisons were performed using Student's t-tests, Mann–Whitney U tests, or chi-square tests as appropriate, with corresponding effect sizes. A linear regression model was used to predict total physical activity from sex and perceived stress.

**Results:**

Men reported significantly higher levels of vigorous (*p* = 0.001) and total physical activity (*p* = 0.001) compared to women, with medium-to-large effect sizes. Moderate-intensity activity was higher in men, but this difference did not reach statistical significance (*p* = 0.056). No significant sex differences were observed in total perceived stress or overall sleep quality. However, women scored higher on two individual PSS-14 items related to perceived lack of control and overload (*p* = 0.013 and *p* = 0.036, respectively). In the regression analysis, sex was a significant predictor of total physical activity (B = −2005.96, standardized *β* = −0.499, *p* = 0.004), whereas perceived stress was negatively associated with activity but did not reach statistical significance (B = −69.76, standardized *β* = −0.267, *p* = 0.104).

**Conclusions:**

Sex differences in physical activity were observed among Kinesiology students, with women reporting lower moderate-to-vigorous activity, while sleep was similar and two individual stress items differed.

## 1 Introduction

Physical, mental, and social well-being is a key component of academic performance and the overall development of university students (1, 2). According to the World Health Organization, health is defined as a state of complete well-being and not merely the absence of disease (3). In the university context, this balance can be influenced by behavioral and psychosocial factors, among which physical activity plays a central role, together with sleep quality and perceived stress (1, 2, 4).

From a theoretical perspective, the relationships among these variables can be understood within the framework of the Transactional Model of Stress and Coping, proposed by Lazarus and Folkman, which conceptualizes stress as a dynamic process resulting from the interaction between the individual and their environment (5, 6). In this model, perceived stress refers to the individual's cognitive appraisal of environmental demands in relation to their perceived coping resources, highlighting its subjective and context-dependent nature (5, 6). This framework provides a useful basis for understanding how perceived stress may influence health-related behaviors and outcomes, including physical activity and sleep quality (5, 6).

Regular physical activity has been consistently associated with better indicators of physical and mental health. In university populations, higher levels of physical activity are related to better sleep quality, lower perceived stress, and greater overall well-being (4, 7, 8). From a theoretical standpoint, physical activity may act as a coping strategy within the stress process, contributing to the regulation of physiological and psychological responses to stress (5, 6). Conversely, sedentary behavior has been linked to mood disturbances, poor sleep quality, and lower academic performance (9–11). Despite its recognized benefits, insufficient physical activity remains prevalent among university students, particularly during periods of high academic demand.

Sleep quality represents another relevant behavioral factor, given its fundamental role in memory consolidation, academic performance, and emotional regulation (12, 13). Sleep quality can be defined as a multidimensional construct that includes subjective satisfaction with sleep, sleep latency, duration, efficiency, disturbances, and daytime dysfunction. However, university students are particularly vulnerable to sleep disturbances due to academic overload, excessive screen time, and psychosocial factors (14, 15). Although 7 to 9 h of sleep per night are recommended, this standard is rarely met (16–18). In Chile, a high prevalence of poor sleep quality has been reported among health sciences students, associated with academic demands and personal problems, leading to daytime dysfunction and mood disturbances (19, 20).

Academic stress is a multifactorial phenomenon that arises when environmental demands exceed available personal resources (21). In line with the Transactional Model, perceived stress specifically reflects the degree to which individuals appraise situations in their lives as stressful, unpredictable, or overwhelming (5, 6). Prolonged exposure to academic stress has been associated with physiological alterations and an increased risk of anxiety and depressive symptoms, particularly among health sciences students due to high academic workload and early exposure to practical and clinical settings (22, 23). Moreover, elevated perceived stress may negatively impact sleep quality through mechanisms such as cognitive hyperarousal and emotional dysregulation, while also reducing engagement in health-promoting behaviors such as physical activity. Conversely, regular physical activity may buffer the negative effects of stress and contribute to improved sleep quality, suggesting a complex and bidirectional relationship among these variables.

The Kinesiology program, focused on the study of human movement and its application in health promotion and functional improvement, constitutes a particularly relevant context for analyzing physical activity patterns and their relationship with sleep quality and perceived stress (24). At the Universidad Catolica de la Santisima Concepcion, a substantial proportion of fourth-year students come from municipalities outside Concepción, which often involves frequent commuting or temporary residence in the city. Research in Chilean university populations indicates that students who live farther from campus tend to engage in less active commuting and present lower overall levels of physical activity; these conditions may be associated with higher stress levels and changes in sleep habits and physical activity behaviors, especially during periods of high academic demand (25).

Although previous studies have documented associations between physical activity, sleep quality, and perceived stress in university populations (1, 2, 4, 7, 8), research simultaneously integrating these three variables in Kinesiology students remains limited. Furthermore, few studies have explicitly examined these relationships within a coherent theoretical framework, which limits the interpretation of the underlying mechanisms linking these constructs. This gap is noteworthy considering the program's focus on health promotion through human movement and the availability of validated instruments at the national level, such as the PSQI, PSS-14, and IPAQ.

Therefore, grounded in the Transactional Model of Stress and Coping, the present study aims to (i) characterize sleep quality, perceived stress, and physical activity levels, and (ii) examine the associations among these variables, with the expectation that higher perceived stress will be associated with poorer sleep quality and lower levels of physical activity, while higher physical activity levels will be associated with better sleep quality and lower perceived stress, in fourth-year Kinesiology students at the Universidad Católica de la Santísima Concepción, Concepción, Chile.

## 2 Methods

### 2.1 Design

This was an observational, descriptive, cross-sectional study, the design of which adhered to the Strengthening the Reporting of Observational Studies in Epidemiology (STROBE) guidelines (26). The study protocol and informed consent procedures were approved by the Ethics Committee of the Universidad Católica de la Santísima Concepción (Protocol Code: 59/2025; Approval Date: October 8, 2025), in accordance with the principles of the Declaration of Helsinki (27).

### 2.2 Context

The study was conducted between October and December 2025 at the facilities of the Universidad Católica de la Santísima Concepción, Concepción, Chile. A non-probabilistic convenience sampling strategy was employed. The target population comprised male and female fourth-year students enrolled in the Kinesiology program at the Universidad Católica de la Santísima Concepción who were registered in at least one fourth-year course, such as Kinesiological Techniques in Cardiorespiratory II, Kinesiological Techniques in Neurology II, Kinesiological Techniques in Musculoskeletal II, Clinical Practice III, or Research Project, and were aged between 21 and 27 years. Data collection was carried out remotely, with questionnaires administered online through a secure electronic (https://forms.gle/WmZDRa5dTLVaDa8o6). Only participants who met the predefined eligibility criteria were included. All participants were fully informed about the objectives of the study and the selection criteria. Subsequently, written informed consent was obtained from all participants, authorizing the use of their data for scientific purposes.

### 2.3 Participants

The final sample consisted of 29 university students (16 women and 13 men), aged between 21 and 27 years, who voluntarily participated in the study. The sample was predominantly composed of students enrolled in clinical and applied courses, reflecting the academic demands characteristic of the fourth year of the program. Inclusion criteria comprised students aged 18 to 30 years who were enrolled in the fourth year of the Kinesiology program at the Universidad Católica de la Santísima Concepción during the 2025 academic year, were actively registered in at least one fourth-year course at the time of data collection, and were able to independently comprehend and complete self-report questionnaires in Spanish. Exclusion criteria included a history of diagnosed chronic conditions (neurological, cardiorespiratory, musculoskeletal, or psychiatric) with potential influence on the study variables, regular use of medications that could affect relevant physiological, cognitive, or perceptual responses, the presence of acute conditions (injury, illness, or significant pain) at the time of assessment, as well as incomplete data, inconsistent responses, or non-compliance with the study protocol.

### 2.4 Sociodemographic and academic variables

Sociodemographic variables included sex assigned at birth, recorded as a categorical variable (male or female) based on self-report, and used to examine sex-related differences to mental health and sleep quality, and age, treated as a continuous variable expressed in years to characterize the sample and enable comparative analyses across age ranges (28). Academic variables included courses enrolled, assessed through self-report of enrollment in fourth-year courses during the academic semester, which was used to confirm eligibility within the target population and to estimate academic workload (29).

### 2.5 Self-report questionnaires

#### 2.5.1 IPAQ-SF

Physical activity levels were assessed using the short version of the International Physical Activity Questionnaire (IPAQ-SF). This instrument comprises seven items designed to evaluate the frequency and intensity of physical activity performed during the previous seven days. Items include questions such as the number of days and duration (in minutes) spent walking, performing moderate activities (e.g., carrying light loads or cycling at a regular pace), and engaging in vigorous activities (e.g., running or high-intensity sports). The IPAQ-SF has been validated in the adult Chilean population (30).

Physical activity was calculated following the IPAQ scoring protocol. Basal metabolic rate (METs) per minute per week was calculated for walking (3.3 METs), moderate activity (4.0 METs), and vigorous activity (8.0 METs), and these were summed to obtain total physical activity. Data were analyzed according to IPAQ data processing guidelines, including truncation of outliers when necessary. Physical activity levels were categorized according to total energy expenditure expressed in MET-minutes per week: low (<600 MET-min/week), moderate (600–1,499 MET-min/week), or high (≥1,500 MET-min/week).

#### 2.5.2 PSQI

The sleep quality was assessed using the PSQI, a self-report instrument validated in Chilean university students (31). The questionnaire consists of 19 items, with a global score ranging from 0 to 21 points, allowing sleep quality to be classified as good sleep quality (0–5 points), poor sleep quality (6–10 points), or severe sleep disturbance (≥11 points). Items assess different dimensions of sleep, including subjective sleep quality, sleep latency (time required to fall asleep), sleep duration, habitual sleep efficiency, sleep disturbances, use of sleep medication, and daytime dysfunction.

#### 2.5.3 PSS-14

The Perceived Stress was assessed using the PSS-14, a self-report instrument been validated in Chilean university population (32). The scale consists of 14 items scored on a 5-point Likert scale (0–4), yielding a total score ranging from 0 to 56, with higher scores indicating greater perceived stress. Total scores allow classification into low perceived stress (0–18 points), moderate perceived stress (19–37 points), and high perceived stress (38–56 points). The instrument evaluates the perception of lack of control and overload in daily life.

### 2.6 Bias

Several potential sources of bias inherent to the study design were considered. First, selection bias may be present due to the use of a non-probabilistic convenience sampling strategy and the voluntary participation of students, which may limit sample representativeness and the generalizability of the findings (33). This limitation is further influenced by the geographic restriction of the sample, as all participants were recruited from a single university located in the city of Concepción, Chile (34). This geographic bias may reduce external validity, since academic, sociocultural, and environmental factors specific to this region could differ from those of students enrolled in other universities or geographic areas. Nevertheless, this risk was partially mitigated by conducting *a priori* sample size calculation. In addition, clearly defined inclusion and exclusion criteria were applied, and the study population was restricted to fourth-year Kinesiology students to enhance sample homogeneity.

Second, the potential for information bias was acknowledged, as key variables were assessed using self-report questionnaires (35). To minimize this risk, standardized and widely used instruments with previously reported psychometric properties were employed, which have been validated or previously applied in Chilean or Latin American populations. Furthermore, data collection was conducted remotely through a secure online platform, ensuring a standardized administration procedure and reducing interviewer-related bias. Finally, the cross-sectional design of the study precludes causal inference and introduces the possibility of reverse causality (36).

### 2.7 Sample size

The sample size was determined by the number of eligible students enrolled in the fourth year of the Kinesiology program during the 2025 academic year (*n* = 36). A total of 29 students agreed to participate, representing approximately 80% of the eligible population.

The sample size estimation was informed by previous studies of sleep quality in Chilean university students. One study reported that 51.8% of students from southern Chile (Patagonia) exhibited poor sleep quality according to the PSQI (37). Assuming a 95% confidence level (Z = 1.96) and a margin of error of 5% (*d* = 0.05), the theoretical minimum sample size for a finite population of 36 eligible students was similar to the number of participants included in this study.

It is important to note that this sample size calculation was intended to support descriptive analyses within the target population. Given the limited sample, the study is primarily descriptive and exploratory; it may be underpowered for detecting small-to-moderate associations in subgroup or regression analyses. Consequently, findings regarding associations (e.g., between stress and physical activity) should be interpreted cautiously, with the understanding that non-significant results may reflect limited statistical power rather than the absence of an effect.

### 2.8 Statistical analysis

Data were analyzed using SPSS version 27 (IBM Corp., Armonk, NY, USA). Continuous variables were summarized as mean ± standard deviation (*SD*) with 95% confidence intervals (CI), while categorical variables were presented as frequencies and percentages. Normality of continuous variables was assessed using the Shapiro–Wilk test. Given the small sample size and potential deviations from normality, descriptive analyses were also inspected using medians (*M*) and interquartile ranges (*IQR*) when appropriate.

For comparisons between two independent groups (men vs. women), the distribution of continuous variables was first assessed using the Shapiro–Wilk test. For normally distributed variables with homogeneity of variances (evaluated using Levene's test), independent-samples Student's t-test were applied (specifically for the total scores of the PSS-14 and PSQI). When the assumptions of normality or homogeneity of variances were not met, comparisons were performed using the nonparametric Mann–Whitney U test (38, 39). Categorical variables were compared using Pearson's chi-square test (40). Appropriate effect sizes were reported for all inferential analyses: Cohen's *d* for t-tests (small: 0.2, medium: 0.5, large: 0.8), *r* for Mann–Whitney U tests (small: 0.1, medium: 0.3, large: 0.5), and Cramér's *V* for chi-square tests (small: 0.1, medium: 0.3, large: 0.5) (41, 42).

A linear regression analysis was conducted to predict total physical activity as measured by the IPAQ-SF. To ensure stability of estimates and avoid overfitting given the limited sample size, only variables that demonstrated significant differences in previous analyses were included as predictors: the total PSS-14 score and sex. The total PSS-14 score was selected because two of its items showed significant differences according to sex, and sex was included to evaluate its independent contribution to physical activity. Other variables, such as “Marital status,” were excluded from the model due to the small sample size (*n* < 30), in line with methodological recommendations to limit the number of predictors and maintain stable and valid regression coefficients (43). This focused approach ensures more reliable inferences from a linear model with few predictors and a limited sample size. Regression results are reported as unstandardized coefficients (B), standardized beta coefficients, 95% confidence intervals, *p*-values, and corresponding effect sizes with conventional classifications. Statistical significance was set at *p* < 0.05.

## 3 Results

### 3.1 Sociodemographic characteristics

 Table 1 summarizes the sociodemographic characteristics and general habits of the sample according to sex. Men (*n* = 13) and women (*n* = 16) showed comparable age and academic workload, with no significant differences between groups (age: 23.15 ± 1.95 vs. 23.13 ± 1.93 years, *p* = 0.948; academic workload: 4.31 ± 1.03 vs. 4.00 ± 1.27, *p* = 0.503). Regarding health status, no diagnosed pathologies were reported among men, whereas 25% of women reported a diagnosed condition, although this difference did not reach statistical significance (*p* = 0.052). A significant difference was observed in marital status, with a higher proportion of women reporting being single compared with men (81.25% vs. 46.15%; *p* = 0.048; effect size = 0.37). No other relevant differences were observed between groups.

**Table 1:** Sociodemographic characteristics and general habits (*n* = 29).

Variables	Men (*n* = 13)		Women (*n* = 16)		Effect size	*p*
	X ± SD	95% CI	X ± SD	95% CI		
Aged (years)	23.15 ± 1.95	21.97 to 24.33	23.13 ± 1.93	22.10 to 24.15	0.02	0.948
Academic workload	4.31 ± 1.03	3.69 to 4.93	4 ± 1.27	3.33 to 4.67	0.14	0.503
Diagnosed pathology (%)	0 (0)		4 (25)		0.36	0.052
No diagnosed pathology (%)	13 (100)		12 (75)			
Marital status single (%)	6 (46.15)		13 (81.25)		0.37	0.048
Marital status couple (%)	7 (53.85)		3 (18.5)			

### 3.2 Physical activity levels

 Table 2 presents the descriptive statistics for individual items and the total score of the IPAQ-SF according to sex. Men (*n* = 13) and women (*n* = 16) showed differences in several physical activity domains. Specifically, men reported significantly higher levels of high-intensity activity compared with women (1,680 ± 1,530.49 vs. 220 ± 436.72 MET-min/week; *p* = 0.001; effect size *r* = 0.62). Moderate-intensity activity also tended to be higher in men (535.38 ± 815.44 vs. 88.75 ± 178.73 MET-min/week), although this difference did not reach statistical significance (*p* = 0.056; effect size *r* = 0.40). Walking activity did not differ significantly between sexes (1,452 ± 1,262.65 vs. 1,064.25 ± 1,205.74 MET-min/week; *p* = 0.351; effect size *r* = 0.18). Total physical activity was significantly greater in men than in women (3,667.39 ± 2,046.23 vs. 1,373 ± 1,364.20 MET-min/week; *p* = 0.001; effect size *r* = 0.59). Finally, sitting time was comparable between men and women (267.69 ± 185.93 vs. 266.25 ± 125.80 min/day; *p* = 1.000; effect size *r* = 0.00). Overall, these findings suggest that men in this sample accumulated higher levels of vigorous and total physical activity than women, whereas walking activity and sedentary behavior were broadly similar between sexes.

**Table 2:** Descriptive statistics and sex differences for individual items and total score of the IPAQ-SF (*n* = 29).

IPAQ-SF		Men (*n* = 13)				Women (*n* = 16)				Effect size	*p*
		SW test	*X* *±* *SD*	M ± IQR	95% CI	SW test	*X* *±* *SD*	M ± IQR	95% CI		
Physical Activity (METs)	High	0.106	1,680 ± 1,530.49	960 **±** 2,400	755.13 to 2,604.87	<0.001	220 ± 436.72	0 ± 360	−12.71 to 452.71	0.62	0.001
	Moderate	<0.001	535.38 ± 815.44	320 **±** 600	42.62 to 1,028.15	<0.001	88.75 ± 178.73	0 ± 120	−6.49 to 183.99	0.40	0.056
	Walk	0.283	1,452 ± 1,262.65	1,188 **±** 2,145	688.99 to 2,215.01	0.011	1,064.25 ± 1,205.74	891 ± 4,158	421.76 to 1,706.74	0.18	0.351
	Total	0.655	3,667.39 ± 2,046.23	3,360 **±** 3,748	2,430.86 to 4,903.91	0.002	1,373 ± 1,364.20	1,194 ± 1,081.50	646.07 to 2,099.93	0.59	0.001
Sitting time (min)		0.130	267.69 ± 185.93	240 ± 240	155.34 to 380.05	0.002	266.25 ± 125.80	300 ± 90	199.22 to 333.28	0	1

### 3.3 Perceived stress

 Table 3 presents the descriptive statistics for individual items and the total score of the PSS-14 according to sex. Men (*n* = 13) and women (*n* = 16) showed comparable scores across most items and for the total PSS-14 score, with no statistically significant sex differences observed at the global level (26.62 ± 9.12 vs. 30.75 ± 6.21; *p* = 0.159; effect size = 0.53). At the item level, two items showed statistically significant differences between sexes. Women reported higher scores than men on item 1 (2.63 ± 0.81, median 3 ± 1 vs. 1.77 ± 0.93, median 2 ± 1; *p* = 0.013; effect size = 0.48) and item 2 (2.69 ± 1.08, median 2.5 ± 2 vs. 1.62 ± 1.33, median 1 ± 2.5; *p* = 0.036; effect size = 0.40). All remaining items showed no statistically significant differences (*p* > 0.05), with effect sizes ranging from very small to moderate (*r* = 0.06–0.37). These results indicate that although some item-level differences were observed, the overall perceived stress score did not differ significantly between men and women in this sample.

**Table 3:** Descriptive statistics and sex differences for score of the PSS-14 (*n* = 29).

PSS-14	Men (*n* = 13)				Women (*n* = 16)				Effect size	*p*
	SW test	*X* *±* *SD*	M ± IQR	95% CI	SW test	*X* *±* *SD*	M ± IQR	95% CI		
Score 1	0.009	1.77 ± 0.93	2 ± 1	1.21 to 2.33	0.036	2.63 ± 0.81	3 ± 1	2.20 to 3.06	0.48	0.013
Score 2	0.161	1.62 ± 1.33	1 ± 2.50	0.82 to 2.42	0.017	2.69 ± 1.08	2.50 ± 2	2.11 to 3.26	0.40	0.036
Score 3	0.035	3.00 ± 1.00	3 ± 2	2.40 to 3.60	<0.001	3.25 ± 0.86	3.50 ± 1.75	2.79 to 3.71	0.12	0.559
Score 4	0.084	1.54 ± 1.05	1 ± 1.50	0.90 to 2.17	0.027	1.63 ± 0.89	2 ± 1	1.15 to 2.10	0.06	0.779
Score 5	0.043	1.39 ± 1.12	1 ± 2	0.71 to 2.06	0.190	1.69 ± 1.08	2 ± 1	1.11 to 2.26	0.14	0.475
Score 6	0.030	1.62 ± 1.33	1 ± 1.5	0.82 to 2.42	0.005	1.94 ± 0.77	2 ± 1.75	1.53 to 2.35	0.22	0.268
Score 7	0.063	1.62 ± 1.04	1 ± 1	0.99 to 2.25	0.002	1.81 ± 0.83	2 ± 1.75	1.37 to 2.26	0.13	0.531
Score 8	0.043	2.62 ± 1.12	3 ± 2	1.94 to 3.29	0.190	2.31 ± 1.08	2 ± 1	1.74 to 2.89	0.14	0.475
Score 9	0.030	1.62 ± 1.33	1 ± 1.50	0.82 to 2.42	0.005	1.94 ± 0.77	2 ± 1.75	1.53 to 2.35	0.22	0.268
Score 10	0.063	1.62 ± 1.04	1 ± 1	0.99 to 2.25	0.002	1.81 ± 0.83	2 ± 1.75	1.37 to 2.26	0.13	0.531
Score 11	0.238	2.15 ± 1.14	2 ± 2	1.46 to 2.85	0.017	1.31 ± 0.95	1.50 ± 1.75	0.81 to 1.82	0.37	0.056
Score 12	0.110	2.23 ± 0.93	2 ± 1.50	1.67 to 2.79	0.040	2.69 ± 1.25	3 ± 2	2.02 to 3.35	0.24	0.232
Score 13	0.271	2.00 ± 1.22	2 ± 2	1.26 to 2.74	<0.001	2.50 ± 0.73	2 ± 1	2.11 to 2.89	0.26	0.199
Score 14	0.039	1.85 ± 1.46	1 ± 2.50	0.96 to 2.73	0.061	2.56 ± 0.96	2.50 ± 1	2.05 to 3.08	0.29	0.132
Score total	0.343	26.62 ± 9.12	25 ± 12	21.10 to 32.13	0.128	30.75 ± 6.21	29 ± 9	27.44 to 34.06	0.53	0.159

### 3.4 Sleep quality

 Table 4 shows the descriptive statistics for each PSQI component and the total score by sex. Overall, men (*n* = 13) and women (*n* = 16) had similar scores across most items and in the total PSQI score, with no significant differences observed at the global level (9.92 ± 3.86 vs. 11.69 ± 3.50; *p* = 0.208; effect size = 0.48). At the level of individual items, none of the PSQI components reached statistical significance (*p* = 0.101–0.812); however, small to moderate effect sizes were noted, particularly for daytime dysfunction (r = 0.34) and usual sleep efficiency (*r* = 0.29), indicating a tendency for slightly higher scores among women. In summary, these findings suggest that sleep quality was generally similar between sexes, with only minor differences in specific domains.

**Table 4:** Descriptive statistics and sex differences for score of the PSQI (*n* = 29).

PSQI	Men (*n* = 13)				Women (*n* = 16)				Effect size	*p*
	SW test	*X* *±* *SD*	M ± IQR	95% CI	SW test	*X* *±* *SD*	M ± IQR	95% CI		
Subjective sleep quality	<0.001	1.69 ± 1.49	3 ± 3	0.79 to 2.60	<0.001	1.94 ± 1.39	3 ± 3	1.20 to 2.68	0.05	0.812
Sleep Latency	0.006	1.92 ± 0.64	2 ± 0.50	1.54 to 2.31	0.004	1.88 ± 1.09	2 ± 1.75	1.30 to 2.46	0.06	0.779
Sleep Duration	0.078	1.85 ± 0.90	2 ± 1.50	1.30 to 2.39	0.004	2.19 ± 0.75	2 ± 1	1.79 to 2.59	0.19	0.351
Usual Sleep Efficiency	0.010	0.85 ± 0.80	1 ± 1.50	0.36 to 1.33	0.025	1.50 ± 1.16	1.50 ± 2.50	0.89 to 2.12	0.29	0.144
Sleep disturbances	<0.001	1.15 ± 0.38	1 ± 0	0.93 to 1.38	<0.001	1.31 ± 0.49	1 ± 1	1.06 to 1.57	0.18	0.475
Use of sleep medication	<0.001	0.62 ± 1.04	0 ± 1.50	−0.02 to 1.25	<0.001	0.50 ± 1.03	0 ± 0.75	−0.05 to 1.05	0.06	0.812
Daytime dysfunction	0.012	1.85 ± 0.80	2 ± 0.50	1.36 to 2.33	0.001	2.38 ± 0.72	2.50 ± 1	1.99 to 2.76	0.34	0.101
Total PSQI Score	0.887	9.92 ± 3.86	10 ± 4.50	7.59 to 12.26	0.132	11.69 ± 3.50	12 ± 3.75	9.82 to 13.55	0.48	0.208

### 3.5 Linear regression analysis

 Table 5 presents the results of a linear regression predicting total physical activity (IPAQ-SF) from perceived stress (PSS-14) and sex. The intercept (constant) indicates an expected IPAQ-SF score of 5,524.06 (95% CI: 3,069.99 to 7,978.14, *p* ≤ 0.001). Sex was a significant predictor, with women showing lower activity levels compared to men (B = −2,005.96, standardized *β* = −0.499, 95% CI: −3,317.82 to −694.09, *p* = 0.004). PSS-14 demonstrated a negative association with total physical activity (B = −69.76, standardized *β* = −0.267, 95% CI: −154.95 to 15.43), although this effect was not statistically significant (*p* = 0.104), suggesting a trend whereby higher perceived stress may relate to lower activity levels.

**Table 5:** Linear regression predicting IPAQ-SF from PSS-14 and sex.

Model		B	Standardized beta	95% CI	*p*
Constant	IPAQ-SF	5,524.064	-	3,069.993 to 7,978.135	≤ 0.001
Predictor	PSS-14	−69.760	−0.267	−154.954 to 15.434	0.104
	Sex	−2,005.955	−0.499	−3,317.818 to −694.093	0.004

## 4 Discussion

The present study aimed to describe sleep quality, perceived stress, and physical activity levels in fourth-year Kinesiology students at a Chilean university, as well as to identify the associations between these variables. The main findings suggest that men reported significantly higher levels of moderate-to-vigorous physical activity compared to women, with moderate-to-large effect sizes, while no statistically significant sex differences were observed in overall perceived stress or sleep quality (although small-to-moderate effect sizes were observed in some comparisons). Additionally, sex was identified as an independent predictor of total physical activity, whereas perceived stress showed a negative, albeit non-significant, association with physical activity levels. Given the exploratory nature of the study and the relatively small sample size, these findings should be interpreted with caution.

One of the most consistent findings of this study was the marked difference in physical activity levels between men and women. Men exhibited significantly higher MET values for moderate and vigorous physical activity, as well as for total physical activity, with statistically significant differences accompanied by medium-to-large effect sizes, suggesting potentially meaningful behavioral differences between sexes within this cohort. Moderate activity showed a non-significant trend towards higher levels in men. These results are generally consistent with previous evidence in university populations, which consistently reports higher participation of men in moderate-to-vigorous physical activity compared to women, both in Chile and internationally (44–46). Beyond these general explanations, these differences may also be interpreted within the Chilean sociocultural context. In Chile, gender norms and socialization processes continue to favor male participation in organized and competitive sports, whereas women may experience greater barriers related to perceived safety, time constraints, and sociocultural expectations surrounding physical activity. Additionally, differences in access to sports spaces and gendered perceptions of exercise may contribute to these disparities (47, 48). The persistence of these differences even among Kinesiology students suggests that academic training alone may not fully counteract broader sociocultural influences, although this interpretation should be approached cautiously.

Interestingly, walking activity and sitting time did not differ significantly between sexes, and the corresponding effect sizes were negligible, indicating that differences were mainly driven by structured or higher-intensity activities rather than by low-intensity or sedentary behaviors. This pattern appears consistent with previous evidence suggesting that women tend to accumulate physical activity through daily tasks or low-intensity movement, whereas men are more likely to engage in planned or vigorous exercise (49, 50).

Contrary to expectations and previous literature, no statistically significant sex differences were observed in total perceived stress scores, although women tended to report higher values with moderate effect sizes in some comparison. In line with the psychometric properties of the instrument, the interpretation of perceived stress was based on total scores rather than item-level analyses. Overall, the levels of perceived stress observed in this sample appear to be moderate, which is consistent with previous findings in university populations (51–53). The absence of significant differences between sexes may be partly explained by the relative homogeneity of the sample, as all participants were fourth-year students exposed to similar academic demands, clinical responsibilities, and evaluation pressures. Such shared contextual factors may have reduced variability in stress levels, thereby limiting the detection of between-group differences (52, 54). Additionally, it is plausible that stress in this population is more strongly influenced by situational academic demands than by sex-related differences.

Overall sleep quality, as assessed by the PSQI, did not differ significantly between men and women, although both groups showed mean scores indicative of poor sleep quality. The absence of statistically significant differences was accompanied by small-to-moderate effect sizes, suggesting that potential differences may exist but were not detected statistically in this sample. This finding is clinically relevant, as it reinforces the notion that sleep disturbances are highly prevalent among university students, particularly in health-related programs characterized by high academic workload and stress (55–57). The lack of significant differences may reflect the strong influence of shared academic and contextual factors, such as workload, irregular schedules, and clinical training demands, which may affect sleep quality similarly across sexes (58, 59). While women tended to report slightly worse sleep quality and higher scores in components such as daytime dysfunction and sleep efficiency, these differences did not reach statistical significance, and therefore should be interpreted cautiously given the limited statistical power of the study.

The lack of strong sex differences in sleep quality contrasts with some studies reporting poorer sleep among female students but aligns with others suggesting that academic stage and workload may be stronger determinants of sleep quality than sex alone. However, the present findings should be interpreted as exploratory and not as definitive evidence, reinforcing the importance of considering contextual and academic factors when interpreting sleep-related outcomes in student populations (55, 60, 61).

The linear regression analysis showed that sex was a significant independent predictor of total physical activity, with women exhibiting substantially lower activity levels than men. Perceived stress showed a negative association with physical activity; however, this relationship did not reach statistical significance. Given the small sample size, the study may have been underpowered to detect associations of small or moderate magnitude. Therefore, the absence of statistical significance should not be interpreted as evidence of absence of association. More broadly, the predominantly non-significant findings observed in this study may indicate that the relationships between physical activity, perceived stress, and sleep quality are complex, potentially bidirectional, and influenced by unmeasured variables (62). The direction of this association is consistent with theoretical models and empirical evidence suggesting that higher stress levels may act as a barrier to physical activity through mechanisms such as fatigue, time constraints, or decreased motivation (63, 64). Nevertheless, these mechanisms were not directly assessed in the present study and should therefore be interpreted cautiously. Additionally, the cross-sectional nature of the study prevents the establishment of causal relationships between the analyzed variables. Consequently, the findings only indicate statistical associations rather than directional or causal relationships. It is plausible that lower physical activity contributes to higher perceived stress, that higher stress reduces participation in physical activity, or that both variables are influenced by external factors such as sleep deprivation, academic overload, or coping strategies (65).

From a practical perspective, the findings may suggest the need to strengthen strategies promoting physical activity among the Kinesiology student population, with particular emphasis on female students, who exhibited lower levels of physical activity. However, given the exploratory nature of the study, these implications should be interpreted cautiously. Interventions addressing gender-related barriers and stress-related constraints could be considered in future research or institutional health programs. Additionally, the high prevalence of poor sleep quality observed in both sexes highlights the importance of incorporating sleep hygiene and recovery strategies into university health promotion programs.

Several limitations should be acknowledged. The small, non-probabilistic sample drawn from a single university limits generalizability. The reliance on self-report instruments introduces the potential for recall and social desirability bias. Additionally, the cross-sectional design prevents causal inference and does not allow assessment of temporal relationships among stress, sleep, and physical activity. The limited sample size may also reduce the statistical power to detect small-to-moderate associations. Furthermore, the lack of statistically significant findings may also be related to restricted variability within a relatively homogeneous sample and to the exploratory nature of the analytical approach. Nonetheless, despite these limitations, the study provides relevant exploratory data and contributes to the still limited evidence specifically focused on fourth-year Kinesiology students in the Chilean context.

Future research should include larger, multicenter samples and longitudinal designs to better understand causal pathways between physical activity, stress, and sleep quality. The inclusion of objective measures of physical activity and sleep (e.g., accelerometry or actigraphy) could further strengthen the evidence. Exploring psychosocial mediators, such as motivation, coping strategies, and academic burnout, may help clarify the mechanisms underlying the associations observed in this exploratory analysis and inform more effective interventions.

## 5 Conclusion

In this sample of Chilean fourth-year Kinesiology students, sex was significantly associated with total physical activity, with women showing lower levels compared to men, both in descriptive analyses and in the linear regression model. Perceived stress showed a negative association with physical activity, although this relationship did not reach statistical significance, while sleep quality was similar between men and women. Overall, these results indicate the presence of sex-based differences in physical activity levels in this population, without showing relevant associations with perceived stress or significant differences in sleep quality. These findings should be interpreted cautiously given the exploratory nature of the study and the limited sample size. The results provide preliminary evidence of sex-related differences in physical activity among Kinesiology students but do not allow definitive conclusions regarding the relationships between stress, sleep, and physical activity.

## Data Availability

The raw data supporting the conclusions of this article will be made available by the authors, without undue reservation.
